# Objective performance and parent-reported executive functioning before and after paediatric transplantation

**DOI:** 10.1192/j.eurpsy.2026.11061

**Published:** 2026-07-31

**Authors:** J. Garrido-Bolton, E. Fernández-Jiménez

**Affiliations:** 1 Department of Psychiatry, Clinical Psychology and Mental Health, La Paz University Hospital; 2 Hospital La Paz Institute for Health Research (IdiPAZ); 3 Department of Child and Adolescent Psychiatry, La Paz University Hospital, Madrid; 4 Faculty of Social Sciences and Communication, Universidad Europea de Madrid, Villaviciosa de Odón (Madrid), Spain

## Abstract

**Introduction:**

A longitudinal neuropsychological assessment is recommended in pediatric patients with serious illnesses before and after solid organ or hematopoietic stem cell transplantation, due to the cognitive deficits they experience, especially in executive functioning. In addition, comparing objective performance with parent-reported performance in executive functioning is essential to adjust expectations about the clinical evolution of patients between both informants.

**Objectives:**

This study aimed to assess objective and parent-reported executive functioning in paediatric patients before and after the transplant.

**Methods:**

A total of 50 patients (54% male) aged between 6 and 18 years (*M* = 11.4, *S.D.* = 3.5) were recruited from La Paz University Hospital (Madrid, Spain). Patients were evaluated both in the pre-transplant phase, while awaiting various types of organ transplants (kidney: 26, heart: 4, lung: 4, hepato-renal: 4, or liver: 2 patients) or haematopoietic stem cells (10 patients), and 6 months after the transplant. Patients were assessed using the *Backward* and *Sequencing* subscales of the *Digit Span* subtest (WISC-V or WAIS-IV version), the *Phonological* and *Excluded-lette *r subscales of the *Verbal Fluency Test*, the *Commissions* score from the K-CPT 2 or CPT 3, and using the Spanish parent-reported version of the *Behavior Rating Inventory of Executive Function*, Second Edition (BRIEF-2) with age- and sex-adjusted norms from the Spanish general population. The paired Student’s t-test or Wilcoxon test was calculated depending on whether the assumption of statistical normality was met. Statistical significance was set at *p* ≤ 0.05.

**Results:**

The only statistically significant change was observed in the *Backward* subscale of the *Digit Span* subtest (Wilcoxon’s Z = -2.05, *p* = .04), which showed an improvement from below-average levels (Mean *z* = -0.74, in the pre-transplant phase) to normal levels (Mean *z* = -0.29, in the post-transplant phase) in relation to the age-adjusted general Spanish population. The other outcomes (objective or reported by parents) showed no statistically significant changes between phases.

**Conclusions:**

Auditory working memory could be a change-sensitive neurocognitive measure for monitoring longitudinal changes in the pediatric transplant population.

**Disclosure of Interest:**

None Declared

